# A Comparative Neuroimaging Review of Repetitive Head Impacts and Mild Traumatic Brain Injury from Pediatrics to Professionals

**DOI:** 10.1177/2689288X251371125

**Published:** 2025-09-05

**Authors:** Natalie M. Bell, Cameron Johnson, Fang F. Yu, Jillian E. Urban, Amy L. Proskovec, Ananth Madhuranthakam, Joseph A. Maldjian, Elizabeth M. Davenport

**Affiliations:** ^1^ Advanced Neuroscience Imaging Research Laboratory, University of Texas Southwestern Medical Center, Dallas, Texas, USA.; ^2^ Department of Radiology, University of Texas Southwestern Medical Center, Dallas, Texas, USA.; ^3^ Department of Biomedical Engineering, University of Texas Southwestern Medical Center, Dallas, Texas, USA.; ^4^ Department of Biomedical Engineering, Wake Forest School of Medicine, Winston-Salem, North Carolina, USA.; ^5^ Wake Forest School of Medicine, Virginia Tech – Wake Forest School of Biomedical Engineering, Winston-Salem, North Carolina, USA.

**Keywords:** concussion, functional neuroimaging, head impact exposure, medical imaging, neuroimaging biomarkers, neurology, pediatric neurotrauma, radiology, subconcussive head impacts

## Abstract

Mild traumatic brain injury (mTBI) or concussion results from a bump or blow to the head followed by clinical symptoms. In contrast, repetitive head impacts (RHI) in sports result from a bump or blow to the head but are not accompanied by acute clinical symptoms. Neither RHI nor mTBI is detectable with traditional neuroimaging. Additionally, pediatric populations remain understudied in this field. Further research in pediatric populations exposed to mTBI/RHI is essential due to ongoing neurological development. This review aims to: (1) evaluate differences in neuroimaging findings in pediatrics and adults, (2) compare differences between RHI findings and mTBI findings within pediatrics, and (3) compare adult and pediatric studies across multiple modalities using the most current neuroimaging literature in the field of pediatric head injury. This narrative review will provide insight into future research directions of the field and potentially inform clinical considerations to be made in the future.

## Introduction

Although mild traumatic brain injuries (mTBI) and repetitive head impacts (RHI) manifest differently, both can cause subtle brain changes that are often overlooked by conventional clinical assessments, highlighting the need for more sensitive tools such as neuroimaging. Concussions-or mTBIs-typically result from a single impact, leading to immediate clinical symptoms,^1–8^ while RHI consists of repeated impacts that accumulate over time without the manifestation of acute clinical symptoms.^9^ However, both can lead to subtle yet significant alterations in the brain that often go undetected by standard clinical evaluations.^9–11^ This issue is further complicated by data suggesting that up to 50% of contact sport athletes may underreport their concussion symptoms to avoid being sidelined.^2,5,12^ Advanced neuroimaging has shown promise in detecting structural and functional brain changes associated with both concussive and RHI exposure, offering a more sensitive approach to diagnosing and monitoring head injuries. Despite their potential, these tools remain underutilized, especially when considering the heightened vulnerability of children and adolescents whose developing brains may experience long-term cognitive and behavioral effects from even seemingly minor head impacts.^10,13–15^

During childhood and adolescence, the brain is in a pivotal phase of growth and maturation, exhibiting a heightened vulnerability to disturbances.^16^ Exposure to RHI may disrupt optimal cognitive development, thereby contributing to learning difficulties and cognitive deficits.^16^ Some research suggests concussion and RHI exposure early in life may have potential long-term consequences, resulting in chronic traumatic encephalopathy (CTE) and other neurodegenerative disorders, underscoring the need for research in this area.^17,18^ Moreover, recent literature has suggested that the cumulative effects of RHI, rather than concussion, may lead to CTE, which is a post-mortem diagnosed neurodegenerative disease characterized by the buildup of abnormal tau protein in the brain.^12,19,20^ Given the association between the cumulative number of head impacts over a lifespan and the development of CTE, it is imperative to develop better concussion diagnostic and RHI management techniques. Specifically, advanced neuroimaging techniques are a promising frontier to aid in the detection of concussion and monitoring the effects of RHI.^21–33^

Traditional neuroimaging, such as computed tomography (CT), has been effective in ruling out severe injuries such as intracranial bleeding or skull fractures. However, CT scans^34^ often fail to detect subtle changes in brain tissue. Additionally, CT uses ionizing radiation, which limits its suitability for certain populations, such as pregnant individuals or young children. Furthermore, CT scans have limited utility in assessing soft tissue injuries.^35,36^ Notably, a retrospective study of individuals aged 10–19 years revealed that 95.4% of CT or magnetic resonance imaging (MRI) findings were non-diagnostic for mTBI.^36^ Structural MRI (e.g., T1-weighted and T2-weighted sequences) studies provide a more comprehensive view of the brain’s structure with high spatial resolution without the use of ionizing radiation.^34^ Though MRI isn’t typically used in the initial stages of concussion diagnosis and management, its ability to create highly detailed images of brain structures makes it an ideal tool for long-term monitoring of brain health and follow-up scans.^37^ However, structural MRIs are unable to detect subtle changes associated with concussion and RHI, making these conditions challenging to identify and monitor in the clinical setting. This limitation highlights the need for more advanced MRI methods.^38^

While structural MRI is adept at revealing anatomical abnormalities, it is less sensitive to the microstructural changes seen with mild concussions and does not track functional changes or cognitive impairments over time. Diffusion imaging techniques (e.g., diffusion tensor imaging [DTI] and diffusion kurtosis imaging [DKI]) offer additional insights beyond structural MRI methods by analyzing the diffusive properties of water in the brain to evaluate structural integrity. This approach provides distinctive and nuanced insights into the intricate microstructural complexities of the brain post-injury.^39,40^ Diffusion imaging exhibits promise to enhance our understanding of concussions and RHI by furnishing detailed information about patterns in microstructural alterations within groups of subjects with similar disease states.

Functional changes are also known to occur in concussion and RHI. Some of the techniques for measuring these changes include functional MRI (fMRI), electroencephalography (EEG), and magnetoencephalography (MEG). fMRI is a neuroimaging technique that measures brain activity by detecting changes in blood flow associated with neural activity. fMRI has shown significant deviations from normal brain regions’ blood flow and neural connectivity, which can reflect blood flow disruption following concussion and RHI.^5,24^ EEG uses electrodes placed on the scalp to record and detect changes in electrical brain activity following concussion and RHI. Abnormalities in brain wave patterns and disruptions in electrical activity can indicate neural dysfunction or injury.^41,42^ MEG is a non-invasive neuroimaging technique that quantifies magnetic fields from neural activity, offering an unparalleled perspective into the dynamic intricacies of brain function at high spatial and temporal resolution.^43–45^ MEG has the potential to detect changes associated with concussion and RHI by measuring disruptions in the brain’s magnetic fields, offering valuable insights into neural activity and connectivity alterations linked to injury.^23,28,32,33,46^ Together, these neuroimaging techniques provide critical insights into the functional alterations associated with concussion and RHI, enhancing our understanding of brain injury mechanisms and aiding in the development of more effective diagnostic and therapeutic strategies.

This review comprehensively examines recently published neuroimaging studies, with a particular emphasis on pediatric populations exposed to RHI and concussive injuries. We assess variations in neuroimaging findings and methodologies reported in the literature for diagnosing, monitoring, and studying mTBI in children and adolescents. This review is intended for clinician-scientists, imaging researchers, and advanced trainees working at the intersection of pediatric neurotrauma and neuroimaging. While we highlight relevant clinical implications, the primary objective is to synthesize recent advancements in advanced neuroimaging techniques used to study mTBI and RHI—particularly in youth. Rather than serving as a clinical management guide, this article aims to bridge translational insights from research imaging modalities to potential future clinical applications. Finally, we compare findings across adult (≥18 years) and pediatric (<18 years) populations, with a focus on identifying critical knowledge gaps in the field.

## Diffusion Tensor Imaging

DTI operates by measuring the diffusion of water molecules in brain tissue. By measuring both the magnitude and directionality of water diffusion, DTI can be incorporated into computational models aimed at quantifying the integrity of white matter tracts.^47^ These computational models can be employed to calculate various metrics, each representing a distinct aspect of brain microstructure. “Tensors” quantify the magnitude and direction of water molecules, while “Eigen values” quantify the rate of water diffusion throughout brain structures. The computation of these metrics facilitates standardization and comparison among subjects.^47–50^ Common metrics include fractional anisotropy (FA), which measures the directionality of water diffusion; mean diffusivity (MD), indicating the overall magnitude of diffusion; axial diffusivity (AD), representing diffusion along the primary axis; radial diffusivity (RD), reflecting diffusion perpendicular to the primary axis; neurite density index (NDI), offering insights into neurite density; and neurite orientation dispersion and density imaging (NODDI), which measures microstructural changes by evaluating volume fraction, orientation dispersion, and isotropic fraction of neurites.^47,51,52^ These metrics collectively provide physiological information about tissue integrity, axonal density, and overall microstructural changes in the brain.^47^

### Adult findings

Analyzing DTI metrics within adult populations has resulted in varied findings. Several studies have noted a decline in DTI metrics, including FA and MD, among individuals exposed to concussion compared to controls.^53–58^ However, the direction of these changes (increased vs. decreased compared to controls) has varied across the reported literature.^55,59–63^ These discrepancies in reported DTI metrics emphasize the necessity for further research and the development of metrics with enhanced sensitivity and specificity. Longitudinal studies have observed mixed responses in DTI metrics during the six-month period post-mTBI as well as after RHI exposure, suggesting that DTI can reflect ongoing recovery from mTBI/RHI but may not serve as a reliable standalone diagnostic tool.^54,55,64–67^

Additionally, significant associations between lower mean FA across white matter tracts and RHI exposure were identified using tract-based spatial statistics (TBSS).^68^ These outcomes suggest the potential of RHI to induce detectable white matter changes in advanced DTI metrics, which could have implications for early detection and monitoring of RHI’s neurobiological impact. However, the directionality of these findings is variable in adults. This variability may be primarily attributed to the timing of the injury, along with the inherently heterogeneous trajectory of concussion recovery.

### Pediatric findings

In pediatric populations, the landscape becomes even more variable, potentially due to the diverse nature of neurodevelopment. Some studies on seasonal contact sports demonstrate reductions in MD/AD/RD and increases in FA following exposure to RHI or subacute/post-acute concussions.^69–73^ Conversely, other studies reported decreased FA in concussion and RHI compared to controls.^58,74–78^ Within the literature, there is inconsistency in pediatric DTI metrics, suggesting variation in the neuropathological response to RHI/mTBI exposure from 24 h post-injury up to 2 weeks post-injury.

More advanced DTI metrics have recently been developed to further quantify and evaluate complex neural architecture by evaluating cellular components.^79^ In one study of pediatric concussion, a positive correlation has been established between NODDI metrics and mTBI symptoms.^80^ However, in a study that evaluated mTBI in pediatrics, DTI and NODDI metrics did not differ between concussed individuals and controls.^71^ Additionally, no published studies were found evaluating RHI in NODDI metrics in pediatrics. A concise overview of the most recent pediatric DTI concussion studies is provided in Table 1.

**Table 1.** Studies Reporting Pediatric DTI Findings in mTBI

Study	Age range	Time since injury	Control type	Group size	DTI analysis	Results
Pinky et al., 2022^70^	12–18 years	Recently concussed: 8 ± 3 days Recovered: 41 ± 16 days	Healthy youth athletes	mTBI (*n* = 45) Control (*n* = 20)	FA, MD, AD, RD Z-scores ANCOVA	MD/AD/RD decreased and increased FA
Shukla et al., 2021^71^	8–17 years	10 days (mean 11 ± 5 days)	OI	mTBI (*n* = 320) Control (*n* = 176)	FA, MD, AD, RD + NODDI ANCOVA with FDR	No significant group differences; age-related changes in FA and MD.
Vasiukova et al., 2021^73^	12–17 years	Acute mTBI (<3 days post-injury)	Healthy Volunteers	mTBI (*n* = 11) Control (*n* = 11)	FA ROI-based	Increased FA
Crasta et al., 2022^76^	12–17 years	<2 wks	Neurotypical peers	mTBI (*n* = 12) Control (*n* = 13)	FA and MD ROI ANOVA.	Increased MD; FA decreased post-recovery.
Ware et al., 2020^63^	8–16.99 years	<24 h	OI	mTBI (*n* = 132) Control (*n* = 69)	FA and MD tractography	No DTI metric group differences. MD positive association with PCS severity at 3 months post-injury.
Stein et al., 2024^80^	8–18 years	1 month and 2–3 months post-mTBI	Healthy controls	mTBI (*n* = 112) Control (*n* = 21)	Voxel-wise NODDI, FA ANOVA	Increased NODDI; NODDI decreased with clinical recovery
Mayer et al., 2022	8–18 years	Sub-acute: 1–11 days post-injury (mean 7.4 ± 2.2) Early-chronic: ≈ 4 months post-injury (131 ± 15 days)	Healthy control	mTBI (*n* = 204) Control (*n* = 173)	Voxel-wise FA, MD, NODDI	Increase FA/NODDI and decreased MD
Lancaster et al., 2016^81^	8–18 years	24 h and 8 days post-concussion	Non-injured athletes	mTBI (*n* = 26) Control (*n* = 26)	Voxel-wise between group FA/MD/AD/RD ANOVA	Decreased MD/AD

This table is a comprehensive list of all the pediatric mTBI studies utilizing DTI.

DTI, diffusion tensor imaging; mTBI, mild traumatic brain injury; FA, fractional anisotropy; MD, mean diffusivity; AD, axial diffusivity; RD, radial diffusivity; NODDI, neurite orientation dispersion and density imaging; ANCOVA, analysis of covariance; FDR, false discovery rate; ROI, region of interest; ANOVA, analysis of variance; OI, orthopedic-injury (control group); PCS, post-concussion symptoms; voxel-wise, whole-brain per-voxel analysis; tractography, diffusion-based reconstruction of white-matter pathways; n, sample size; h, hours; wks, weeks.

The variability of findings in literature underscores the ongoing challenges in establishing standardized criteria for both pediatric and adult populations. This could be due to the need for significantly larger sample sizes when investigating heterogeneous conditions like mTBI and RHI exposure. Alternatively, it may indicate that personalized approaches to monitoring and diagnostics are required in these cases. Furthermore, explicitly reporting time from injury, time elapsed from RHI exposure, overall RHI measurement techniques (sensor type-helmet vs. mouthguard, recording threshold, etc.) may have an impact on these metrics and should be reported in these neuroimaging studies. Additionally, establishing a consensus within the scientific community on standardized pre-processing steps for evaluating changes in DTI metrics could help address the variability in results reported here. Lastly, DTI data is limited to assessing the Gaussian distribution of cellular-level changes within brain tissue, which may restrict its effectiveness in evaluating mTBI and RHI exposure in both adults and pediatric populations. Future research should investigate the non-gaussian diffusion, or temporal dynamics across the brain tissue of those affected by mTBI and RHI.

## Diffusion Kurtosis Imaging

DKI is another form of diffusion imaging that explores brain tissue microstructures by measuring water molecule deviation from a Gaussian diffusion pattern. Differing from DTI, DKI employs a broader range of b-values, exceeding 1000 s/mm^2^, capturing anisotropic and non-Gaussian diffusion patterns crucial for insights into tissue microstructure complexities.^82,83^ Diffusional kurtosis estimation (DKE), tractography, and TBSS refine DKI data by incorporating non-Gaussian kurtosis metrics.^84^ This improves sensitivity and specificity in detecting microstructural changes compared to conventional DTI, which only measures Gaussian diffusion, making these techniques more effective for identifying subtle abnormalities and tissue heterogeneity.^85,86^ Many metrics can be calculated, including mean kurtosis (MK), which reflects the organization of microstructure within the brain tissue, axial kurtosis (AK), which measures the intricacies of the microstructure along certain directional pathways within the tissue being examined, and radial kurtosis (RK), which measures the complexity, organization, or potential abnormalities of structures perpendicular to the principal diffusion direction.^87^ These metrics differ from traditional diffusion measures, offering valuable information for characterizing tissue microstructure in research and clinical applications. DKI’s heightened sensitivity to both the heterogeneity and complexity of the tissue microenvironment distinguishes it from DTI.^82,83^

### Adult findings

Like DTI, variable findings have been reported when using DKI to evaluate concussion and RHI exposure in adult populations. There was an association found between cumulative RHI exposure (number of years of contact sports) and increased FA and RK,^62^ while other studies reported lower MK, AK, and RK among adult mTBI groups in comparison to controls.^88–90^ In contrast, some groups have reported increased MK and AK measures post-concussion.^81,91^ Many authors highlight the limitations of these studies, particularly the scarcity of longitudinal data. However, there are few longitudinal studies published to date that extend over 60 days. One longitudinal study utilized DKI in TBSS to evaluate recovery of both high school and college-aged, concussed athletes. At multiple time points (<48 h, 8 days, and 15 days), participants exposed to RHI had increased AK when compared to controls. However, in that same study the RHI group AK returned to baseline in 45 days.^92^

### Pediatric findings

Evaluation of RHI and concussion effects among pediatric patients showed increased MK, RK, and AK in RHI individuals compared to controls.^81,92,93^ In evaluating RHI effects among pediatric subjects, DKI metrics showed decreased MK values.^94^ DKI-MK demonstrated a significant correlation with RHI exposure, indicating its potential as a reliable indicator in this context.^22^ The variable DTI findings (increased and decreased FA, MD, AD, RD) within pediatric RHI and concussion groups make DKI potentially a more effective modality when evaluating pediatric groups. A consolidated summary of the pediatric DKI concussion studies, including key metrics and outcomes, is provided in Table 2.

**Table 2.** Studies Reporting Pediatric DKI Findings in mTBI

Study	Age range	Time since Injury	Control type	Group size	DKI analysis	Results
Lancaster et al., 2016^81^	8–18 years	24 h and 8 days post-concussion	Non-injured athletes	mTBI (*n* = 26) Control (*n* = 26)	Voxel-wise between group AK ANOVA	Increased AK
Muftuler et al., 2020^92^	16–20 years	<48 h, 8 d, 15 d, 45 d post-injury	Non-injured American football athletes	mTBI (*n* = 96) Control (*n* = 82)	TBSS—Voxel-wise KFA/KAX/KRD	Increased KAX (<48 h, 8 day); Decreased KFA/KRD (15–45 day)

This table lists all the pediatric mTBI studies utilizing DKI.

DKI, diffusion kurtosis imaging; MK, mean kurtosis; KAX, axial kurtosis; KRD, radial kurtosis; KFA, kurtosis fractional anisotropy; TBSS, tract-based spatial statistics; d, days.

The application of DKI has shown promise in identifying sustained alterations in brain structure, potentially offering a valuable tool for tracking recovery and leading to a more informed and personalized approach to concussion care. While pediatric findings with DKI are relatively consistent, reporting increases in DKI metrics—primarily MK—after concussion and RHI exposure, adult findings with DKI are more variable. This variability is like that observed in adult DTI studies and highlights the need for further research to better understand the underlying causes of adult DKI findings. Potential limitations of DKI include its sensitivity to different injury mechanisms, stages of recovery, and individual differences. Establishing standardized protocols and addressing these variability issues will be crucial for maximizing the clinical utility of DKI in both pediatric and adult populations.

A primary limitation of diffusion MRI (including DTI and DKI) is its susceptibility to patient movement, making it particularly challenging for populations such as children or individuals with cognitive impairments due to the extended duration and noise levels of the acquisition. This may be a constraint of the existing literature, as few studies have focused on pediatric populations. Despite these challenges, DKI has shown promise in detecting more consistent findings in pediatric RHI and concussed individuals compared to DTI.

### fMRI

fMRI is a technique that measures changes in blood oxygen level-dependent (BOLD) signal as an indicator of neural activity.^95,96^ fMRI examines brain function by measuring changes in cerebral BOLD signal during specific tasks or at rest.^96^ Thus, this method identifies active brain regions and allows for the analysis of brain network connectivity.^97^ fMRI has been used to study a variety of conditions, ranging from concussions to neurodegenerative disorders and psychiatric illnesses.^96,97^ This section of the review focuses primarily on findings from resting-state fMRI literature.

### Adult findings

fMRI has been used in multiple studies of concussion and RHI exposure in adults, showing changes across connectivity, neural signatures, and associated changes between fMRI and neurocognitive health. Group differences between mTBI participants and controls have been observed in lower-frequency spectral power, a pattern typically associated with sleep in healthy individuals.^98,99^ This suggests that alterations in low-frequency spectral power in resting-state fMRI may serve as a biomarker for mTBI. There have been multiple studies of resting-state fMRI functional connectivity (rsFC) metrics in adult mTBI patients. Studies have reported decreased connectivity in attention and executive control networks in mTBI patients compared to controls.^100–102^ Additionally, rsFC has been shown to have a positive association with the number of previous concussions, suggesting cumulative effects on brain network function reflected in these measures.^53,54,103,104^ Furthermore, rsFC metrics have also been significantly associated with neuropsychological metrics in the context of concussion.^100,104^ Within the concussion literature there are hypoconnectivity and hyperconnectivity that are associated with cognitive processing, attention, and clinical presentations.

When evaluating RHI exposure, a similar effect is observed, with rsFC showing increased activity in cortical regions.^105–107^ This suggests that both concussion and RHI exposure may be resulting in similar connectivity patterns, which supports the notion that both RHI and concussion contribute to later-in-life disorders.^12^

However, some studies have found inconsistencies in the ability of fMRI to detect changes related to concussion and RHI. In a retrospective study that evaluated repetitive concussion and head impact exposure in former collegiate athletes (36–41 years), neither concussion history nor the RHI metric used in the study was correlated with fMRI neural signatures.^108^ However, the same study evaluated fMRI connectivity and found that these rsFC metrics were associated with RHI in regions previously implicated in pathological aging.^108^ It is important to note that the RHI metric used was a head impact exposure estimate calculated from football playing history. The head impact field is an evolving and emerging field that will, parallel with imaging advancements, improve our understanding of the effects of RHI on the brain. These findings altogether reveal potential use of fMRI within RHI monitoring; however, these findings are not consistent across the adult population.

### Pediatric findings

Studies using fMRI to evaluate concussion and RHI in pediatric populations have shown differing functional connectivity patterns (primarily increased) of pediatric subjects exposed to RHI or concussion when compared to a control group. Primarily, differences were seen across the default mode network (DMN), central executive network (CEN), and the salience network (SN).^109–112^ These findings may inform various treatment pathways in the future for clinicians to target post-concussion. Additionally, a longitudinal cohort study examined potential abnormalities in resting-state fMRI neural signatures following pediatric mTBI and revealed increased fMRI metrics in the mTBI participants compared to healthy controls at both 1 week and 4 months post-injury. However, no significant group differences were found in either metric between patients with persistent post-concussive symptoms and recovered patients or controls at 4 months post-injury.^113^ These findings suggest that fMRI neural signatures may be a sensitive marker for acute changes following mTBI but may not differentiate between persistent symptoms and recovery in the later stages when looking at pediatrics. A comprehensive overview of the most recent pediatric concussion studies that employed fMRI—including analytic approaches and key connectivity findings—is presented in Table 3.

**Table 3.** Studies Reporting Pediatric Resting-State fMRI Findings in mTBI

Study	Age range	Time since Injury	Control type	Group size	fMRI analysis	Results
Vaughn et al., 2022^109^	8–15 years	7 weeks post-injury	Healthy controls	mTBI (*n* = 70) Control (*n* = 50)	Rs-fMRI BVAR-FC	Increased FC; magnitude varies with injury severity and predicts later PCS/anxiety
Healey et al., 2022^110^	10–18 years	4 weeks post-injury	Orthopedic injury	mTBI (*n* = 55) Control (*n* = 27)	rs-fMRI seed-based FC	Increased CEN and SN connectivity; decreased DMN-CEN connectivity
Iyer et al., 2020^111^	8–18 years	1 month post-injury	Non-contact athlete	mTBI (*n* = 62)	Whole-brain resting-state functional-connectivity	Increased posterior DMN FC
Stephenson et al., 2020^113^	8–18 years	1 week and 4 months post-injury	Non-contact athlete	mTBI (*n* = 105) Control (*n* = 113)	Resting-state fALFF & ReHo	Increased striatal activity; ReHo: No significant group differences

This table is a comprehensive list of all the pediatric mTBI studies utilizing resting-state fMRI.

rs-fMRI, resting-state functional MRI; BVAR-FC, Bayesian vector-autoregressive functional connectivity; FC, functional connectivity; CEN, central executive network; SN, salience network; DMN, default mode network; fALFF, fractional amplitude of low-frequency fluctuations; ReHo, regional homogeneity; seed-based, ROI-seeded connectivity analysis.

In addition to concussion work in fMRI, evaluation of longitudinal changes in fMRI activity differs between the RHI groups and the control groups.^114^ Longitudinal cerebrovascular (fMRI) changes occur within a season of contact sport play, associated with accumulated RHI exposure.^24,114^ This highlights the need to monitor the progression of RHI exposure in pediatric athletes across contact sports from season to season. This compounds with the assumption that RHI exposure is thought to increase concussion risk as well as increased risk of neurological disorder development later in life.^16,19,115–119^

When comparing findings from fMRI studies of concussion and RHI in adults and pediatrics, several similarities and differences become apparent. Similarities include the potential of multiparameter combinations of resting-state fMRI metrics to improve the classification of mTBI patients from normal controls, underscoring fMRI’s diagnostic relevance in both age groups. Additionally, both adult and pediatric populations exhibit differing functional connectivity patterns (primarily increased) in response to RHI or concussion, indicating shared alterations in brain networks. However, differences begin to emerge, with studies in adults failing to establish associations between lifetime head trauma exposure and fMRI neural signatures, while pediatric studies reveal significantly different relationships between cognition, RHI/mTBI, and fMRI connectivity compared to control groups. Both adult and pediatric studies share concerns regarding the effects of RHI on cerebral blood flow, as evidenced by differing longitudinal changes in cerebrovascular activity. In summary, while there are commonalities in the vascular changes observed in fMRI due to concussion and RHI, nuanced differences emphasize the need for age-specific considerations in understanding these effects.

### EEEG

EEG is a neurophysiological technique used to record the brain’s electrical activity.^120^ EEG differs from fMRI in both its acquisition methodology and the biological signals it measures (i.e., electrical neural current). Instead of measuring cerebrovascular brain function as in fMRI, EEG measures electrical activity at the skin surface. By placing electrodes on the scalp, it detects and measures voltage fluctuations generated by synchronized neuronal activity, offering high temporal resolution for studying neural oscillations, event-related potentials, and connectivity.^120–122^ EEG commonly examines six canonical frequencies that are seen with normal brain activity [delta (1–4 Hz), theta (4–7 Hz), alpha (7–12 Hz), beta (15–30 Hz), low gamma (30–80 Hz), and high gamma (80–150 Hz)].^123^ Power spectral density (PSD) is a measure that quantifies the distribution of signal power across different frequency components, helping to analyze the intensity of specific frequencies in a signal.^124^ PSD can be used to identify pathological dysfunction. In concussion research, EEG monitors electrical brain activity after injury, revealing markers of concussion severity, recovery trajectory, and providing insights into cognitive function post-concussion.^125^ Factors like post-traumatic amnesia, loss of consciousness, cognitive performance, and combat experience can influence long-term EEG changes following concussion.^126^ Overall, EEG serves as a valuable tool in both clinical and research settings for monitoring brain activity and identifying neurological dysfunction, especially in the context of concussion.

### Adult findings

Research on EEG in adults with concussion has provided insights into its potential diagnostic and prognostic applications, especially in the context of mTBI. Notably, EEG power changes have been significantly correlated with injury characteristics, cognitive issues, and psychological distress, all of which are associated with negative long-term outcomes following mTBI.^126–128^ Concussion symptoms can persist for 4–6 weeks post-injury. Some studies have observed decreases in EEG measures during the acute phase (<24 h post-injury) and increases during the subacute phase (4–6 weeks post-injury) of concussion.^128^ However, EEG readings during these phases have been reported as elevated (alpha power) when compared to controls.^129^ In conjunction with EEG power alterations, both mouse and human studies have suggested that EEG slow waves and global connectivity measures during wakefulness are potential markers for mTBI.^42^

The brain function index (BFI) was created from EEG data as an artificial intelligence diagnostic tool for mTBI that evaluates quantitative EEG measurements.^130^ The BFI takes relative power, total power, and connectivity measures (coherence, phase synchrony, etc.) into account in classifying the severity of traumatic brain injury (TBI).^131^ This algorithm was trained using 2000 TBI subjects (mild to severe TBI) and almost 400 controls.^130,131^ Additionally, the BFI algorithm is based on data from adult populations. These findings suggest that EEG can effectively assess and monitor neurophysiological changes in concussed adults. However, future studies may explore the EEG–BFI algorithm in pediatric populations as well as in RHI monitoring efforts.

EEG assessment within the context of RHI-exposed adults is still largely understudied and reported on. In the few studies that focused on resting-state EEG and RHI exposure, alterations in EEG were found in former NFL football players using the EEG BFI.^132^ This study did not report on the time elapsed since participants’ last played football, but these EEG findings were compared to a non-RHI-exposed control group and to the age of first exposure. Most importantly, this study found that the BFI score was associated with clinical features (executive function, memory, behavior, etc.).^132^ Outside of the BFI literature, 18–23 year-old water polo athletes showed increased slow-wave connectivity when comparing pre-season to post-season EEG recordings.^133^ These studies align with findings in concussed adults, demonstrating the cumulative effects of RHI exposure on the brain.

### Pediatric findings

Pediatric EEG differs significantly from adult EEG patterns in typically developing individuals as well as across many different diseases.^134–136^ As such, pediatric subjects might exhibit a different set of electrophysiological markers that denote injury, recovery, or both. Understanding these differences is critical for age-specific interventions and therapeutic strategies. One study observed differences in brain connectivity between healthy adolescents and those recently concussed, suggesting a functional restructuring of resting state networks after concussion (<1 week).^137^ Similarly, when analyzing adolescent athletes, combining resting-state EEG with graph theoretical approaches revealed marked alterations in local brain network properties following a concussion.^138^ Connectivity measures can be a valuable tool to assess post-injury brain network changes and identify those at increased risk for subsequent injuries.^138^ Moreover, a comprehensive study involving 354 male contact sport athletes showcased the EEG–BFI as a distinguishing tool for concussed athletes, especially within 72 h post-injury. Interestingly, BFI metrics reverted to control levels by day 45, reinforcing its value as a potential objective indicator for both concussion identification and recovery monitoring in both adults and pediatrics.^139^ However, the BFI metric has limitations, as it does not appear to effectively distinguish between RHI and concussion groups. Some studies focused on basic attention-related EEG measures, finding differences between pre-season and post-season recordings as well as associated EEG changes with head impact exposure.^140^ Conversely, subconcussive events-when recurrent-may induce nuanced, incremental EEG shifts.^140^ A concise synthesis of pediatric concussion EEG studies can be found in Table 4.

**Table 4.** Studies Reporting Pediatric Resting-State EEG Findings in mTBI

Study	Age range	Time since Injury	Control type	Group size	EEG analysis	Results
Hristopulos et al., 2019^137^	15–16 years	≤1-week post-injury	Non-contact athlete	mTBI (*n* = 21) Control (*n* = 32)	EEG-based effective connectivity	Increased frontal-temporal FC; decreased midline
Virji-Babul et al., 2014^138^	15–18 years	Clinically diagnosed concussion	Non-contact athlete	mTBI (*n* = 9) Control (*n* = 33)	Resting-state EEG with graph theoretical analysis using Bayesian network modeling	Increased frontal hubness and decreased frontopolar degree
Brooks, 2018^139^	14–22	Baseline; <72 h; 45 d post-injury	Contact athlete	mTBI (*n* = 110) Control (*n* = 244)	Resting-state EEG machine learning from frontal/frontotemporal leads	Decreased machine learning metrics at 72 h time point

This table is a comprehensive list of all the pediatric mTBI studies utilizing resting-state EEG.

EEG, electroencephalography; rs-EEG, resting-state EEG; FC, functional connectivity; effective connectivity, directed influence between brain regions inferred from time-series; graph-theoretical analysis, network metrics computed from connectivity matrices; degree, number of connections for a node; hubness, node centrality/prominence; frontopolar, prefrontal pole region; Bayesian network modeling, probabilistic graphical modeling of directed connections; ML, machine learning; BFI, Brain Function Index (ML-derived EEG score; used in some studies).

Overall, EEG in pediatrics has reported similar results to those of adults in finding elevated slow-wave EEG in both concussion and RHI. However, there is still more work to be done focused on EEG changes in RHI exposure in pediatrics. There are some limitations when it comes to EEG that must be considered. EEG’s inherent spatial resolution constraints might inhibit detailed insights into activity in specific regions. EEG is prone to artifacts, both biological (e.g., ocular movements) and external.^120^ Proper EEG interpretation demands expertise, with the risk of missing subtle shifts without in-depth analysis or sophisticated computational methods. Overall, the EEG findings comparing adults to pediatrics are somewhat consistent with each other, like what was seen in fMRI, which may indicate that functional neuroimaging may be the most consistent modality for concussion diagnostics.

### MEG

MEG is a noninvasive functional neuroimaging technique that has gained significant attention for its utility in assessing individuals with TBI and RHI. MEG offers superior spatial and temporal resolution compared to other functional neuroimaging modalities by measuring magnetic fields from post-synaptic dendritic currents, which enable these signals to be source localized.^43^ It allows researchers and clinicians to pinpoint brain activity with remarkable precision. Unlike some other neuroimaging techniques (such as fMRI, which indirectly measures brain activity through changes in blood flow), MEG directly measures neuronal activity.^44,45^ MEG also has a high sampling rate, allowing it to capture brain activity at a significantly higher rate than fMRI. While EEG shares a similar temporal resolution with MEG, it relies on measuring the electrical signals generated by neurons. Unfortunately, the electrical fields can be susceptible to distortion caused by variations in the electrical properties of tissue like the skull and skin, resulting in reduced spatial resolution.^44,120^ MEG enables a more refined spatial and temporal assessment of neural activity while remaining non-invasive, making it an ideal choice for studying dynamic changes in the brain following TBI or RHI.

### Adult findings

Research on MEG measures has provided some insight into potential diagnostic applications, especially in the context of mTBIs within adult populations.^141–144^ An overall increase in low-frequency power (delta, theta, alpha) was seen in adult mTBI groups when compared to healthy and cognitively normal adults, potentially highlighting MEG diagnostic capabilities.^141,145–148^ Additionally, these low-frequency MEG measures have been associated with clinical variables (Delis-Kaplan Executive Function System & Wechsler Adult Intelligence Scale).^149^

Broadly, hyper-connectivity and hypo-connectivity measures have been detected in MEG metrics in adults. Increased delta, theta, alpha, and beta resting-state connectivity was documented when comparing mTBI groups within 3 months or less of injury to control groups.^145,146^ However, some studies have reported reduced connectivity across the brain in mTBI participants compared to controls in both regional power and functional connectivity,^148,150^ and connectivity measures have been correlated with neuropsychological measures as well.^151–154^ With the connectivity measures showing both increases and decreases in certain networks, the understanding is that this may be a compensatory response.

When evaluating concussion recovery (at least 6 months post-injury), one study reported increases in local connections in the follow-up MEG scan (1 year post-injury) when compared to the baseline resting-state MEG scan.^152^ In individuals with combat-related mTBI, heightened resting-state gamma activity- potentially indicating GABA-ergic interneuron dysfunction was observed throughout various brain regions and associated with cognitive deficits, suggesting its potential as a biomarker for mild head injuries.^30^ Another study demonstrated elevated theta-band power following mTBI.^46^ In adults, various techniques have proven useful in distinguishing between unaffected individuals and those with TBI.

### Pediatric findings

Overall, when evaluating pediatric concussion with MEG, low-frequency band power was reported during instances of concussion. Across studies, increased delta power and delta-band effective connectivity were the most observed changes. Two studies by Huang et al. (2020, 2023) using Fast-VESTAL showed that delta and gamma bands were most predictive of mTBI and that brain activity changes were spatially variable across frequency bands. Davenport et al. (2022) reported increased delta power post-season, and Reddy et al. (2021) identified significant increases in delta-band effective connectivity, suggesting persistent functional alterations. Overall, delta-band abnormalities appear to be a reliable marker of pediatric mTBI in MEG studies. Table 5 outlines the pediatric concussion studies found.

**Table 5.** Studies Reporting Pediatric Resting-State MEG Findings in mTBI

Study	Age range	Time since Injury	Control type	Group size	MEG analysis	Results
Huang et al., 2023^33^	8–15 years	3 weeks post-injury	Orthopedic Injury	mTBI (*n* = 59) Control (*n* = 39)	Fast-VESTAL Machine Learning	Increased delta and gamma bands
Davenport et al., 2022^22^	14–18 years	Baseline, <72 h post-concussion, 4 month post-baseline	Noncontact athletes & Non-concussed Contact athletes	mTBI (*n* = 8) Control (*n* = 16)	Difference Maps ANOVA	Increased delta power post-season.
Reddy et al., 2021^28^	14–18 years	Baseline, <72 h post-concussion, 4 month post-baseline	Noncontact athletes & Non-concussed Contact athletes	mTBI (*n* = 8) Control (*n* = 16)	Effective Connectivity ANOVA	Increases in effective connectivity significant in delta band.
Huang et al., 2020^31^	8–15 years	6 months post-mTBI	Orthopedic Injury	mTBI (*n* = 12) Control (*n* = 12)	Fast-VESTAL Voxel-wise t-tests	Hyper- and hypoactivity variable across bands

This table is a comprehensive list of all the pediatric mTBI studies utilizing resting-state MEG.

MEG, magnetoencephalography; rs-MEG, resting-state MEG; Fast-VESTAL, fast vector-based spatio-temporal analysis using L1-minimization; delta band, low-frequency oscillations (∼1–4 Hz); gamma band, high-frequency oscillations (∼30–80+ Hz); SVM, support vector machine (machine-learning classifier).

Existing studies suggest that certain brain activity patterns-especially in the low-frequency bands-are key indicators of concussions in children. MEG has provided detailed insights for adult and pediatric TBI assessments. Much like EEG, there is still limited literature on pediatric RHI exposure. More research is needed to determine similarities between concussion and RHI in MEG, especially given the potential long-term implications of concussions on children’s future mental health.

The primary limitation of MEG is its limited availability, which would restrict widespread clinical and research applications. However, the availability of MEG as a neuroimaging modality has increased recently. This expansion in accessibility means that more researchers and clinicians have access to this powerful tool, allowing for a wider range of applications and contributing to a better understanding of brain function and dysfunction in mTBI and RHI cases. In addition to more widespread use, optically pumped magnetometers (OPM)-or “mini” MEG systems-are growing in popularity for their affordability, mobility, and lack of need for cryogens.^155^ OPMs like EEG allows for some movement, making the modality more suitable and easier to use with children.^120,156^

Recent studies have shown an association between RHI exposure and CTE, highlighting the need for further research on pediatric neuroimaging in the context of RHI. Additionally, integrating functional neuroimaging findings with other neuroimaging modalities and clinical assessments could provide a more consistent and comprehensive understanding of the effects of RHI on brain health. Such research could inform the development of targeted interventions and preventative strategies to mitigate the long-term consequences of RHI.

## Conclusion

Emerging trends in neuroimaging appear promising for improving the diagnosis, monitoring, and management of concussion and RHI. Although conventional imaging is still used chiefly to exclude more serious injuries, the studies discussed in this review suggest that newer, more sensitive modalities may detect subtler brain changes. Such advances could support more individualized treatment strategies, better-informed return-to-play decisions, and more data-driven safety guidelines in contact sports. Over time, neuroimaging may become an important component of both concussion care and broader RHI surveillance and policy development.

Among the most promising trends are the use of DKI in pediatric populations, which has demonstrated more consistent findings than traditional DTI; the application of resting-state MEG and EEG to detect low-frequency abnormalities following RHI or concussion; and machine learning—based classification techniques that may improve diagnostic specificity. Additionally, the integration of multimodal imaging data (e.g., combining functional and structural metrics) and longitudinal imaging studies following concussion or RHI is emerging as a key approach to better understand the temporal evolution and cumulative effects of head impacts. However, across all modalities, further research and development of larger normative databases for these advanced metrics are critical to fully understand the clinical utility of these methods.

As a narrative review, we did not apply standardized inclusion criteria for the timing of imaging post-injury or for specific pediatric age subgroups across modalities. When such details were clearly reported in the literature, they were noted; however, variability in reporting limited consistent analysis of these variables across studies.

While this review is primarily intended for researchers and trainees, the findings may support future clinical guidelines as imaging tools become more widely validated. By comparing neuroimaging biomarkers across age groups and injury types, we aim to foster dialogue between clinicians, scientists, and developers of next-generation diagnostic tools.

## Transparency, Rigor, and Reproducibility

This study was not formally registered because it is considered a narrative review. The analysis plan was not formally registered. No formal statistical analysis was performed on this narrative review. Pubmed, Web of Science, and Google Scholar were used, and keywords included terms such as concussion, pediatric concussion, head impact exposure, and repetitive head impact exposure. Additionally, the most recent journal publications were prioritized. No replication or external validation studies have been performed or are planned/ongoing at this time to our knowledge. There is no analytic code associated with this study.

## Abbreviations Used

AD: axial diffusivity
AK: axial kurtosis
BFI: Brain Function Index
BOLD: blood oxygen level–dependent
CEN: central executive network
CT: computed tomography
CTE: chronic traumatic encephalopathy
DKE: Diffusional Kurtosis Estimator
DKI: diffusion kurtosis imaging
DMN: default mode network
DTI: diffusion tensor imaging
EEG: electroencephalography
FA: fractional anisotropy
fMRI: functional magnetic resonance imaging
GABA: gamma-aminobutyric acid
MD: mean diffusivity
MEG: magnetoencephalography
MK: mean kurtosis
MRI: magnetic resonance imaging
mTBI: mild traumatic brain injury
NDI: neurite density index
NODDI: neurite orientation dispersion and density imaging
OPM: optically pumped magnetometer
pCASL: pseudo-continuous arterial spin labeling
PSD: power spectral density
RD: radial diffusivity
RHI: repetitive head impacts
RK: radial kurtosis
rsFC: resting-state functional connectivity
SN: salience network
TBI: traumatic brain injury
TBSS: tract-based spatial statistics
